# Weighted gene coexpression correlation network analysis reveals the potential molecular regulatory mechanism of citrate and anthocyanin accumulation between postharvest ‘Bingtangcheng’ and ‘Tarocco’ blood orange fruit

**DOI:** 10.1186/s12870-023-04309-5

**Published:** 2023-06-02

**Authors:** Yan Jin, Manyu Liao, Na Li, Xiaoqian Ma, Huimin Zhang, Jian Han, Dazhi Li, Junfeng Yang, Xiaopeng Lu, Guiyou Long, Ziniu Deng, Ling Sheng

**Affiliations:** 1 National Center for Citrus Improvement Changsha, College of Horticulture, Hunan Agricultural University, Changsha, CS China; 2 Hunan Horticultural Research Institute, Changsha, CS China

**Keywords:** Transcriptome, Anthocyanin, Citrate, ‘Bingtangcheng’ sweet orange, ‘Tarocco’ blood orange, Postharvest

## Abstract

**Background:**

Organic acids and anthocyanins are the most important compounds for the flavor and nutritional quality of citrus fruit. However, there are few reports on the involvement of co-regulation of citrate and anthocyanin metabolism. Here, we performed a comparative transcriptome analysis to elucidate the genes and pathways involved in both citrate and anthocyanin accumulation in postharvest citrus fruit with ‘Tarocco’ blood orange (TBO; high accumulation) and ‘Bingtangcheng’ sweet orange (BTSO; low accumulation).

**Results:**

A robust core set of 825 DEGs were found to be temporally associated with citrate and anthocyanin accumulation throughout the storage period through transcriptome analysis. Further according to the results of weighted gene coexpression correlation network analysis (WGCNA), the turquoise and brown module was highly positively correlated with both of the content of citrate and anthocyanin, and p-type ATPase (*PH8*), phosphoenolpyruvate carboxylase kinase (*PEPCK*), chalcone isomerase (*CHI*), flavanone 3-hydroxylase (*F3H*), flavonoid 3’-hydroxylase (*F3’H*) and glutathione S transferase (*GST*) were considered key structural genes. Moreover, MYB family transcription factor (*PH4*), Zinc finger PHD-type transcription factor (*CHR4*, *HAC12*), Zinc finger SWIM-type transcription factor (*FAR1*) and Zinc finger C3H1-type transcription factor (*ATC3H64*) were considered hub genes related to these structural genes. Further qRT-PCR analysis verified that these transcription factors were highly expressed in TBO fruit and their expression profiles were significantly positively correlated with the structural genes of citrate and anthocyanin metabolism as well as the content of citrate and anthocyanin content.

**Conclusions:**

The findings suggest that the CHR4, FAR1, ATC3H64 and HAC12 may be the new transcription regulators participate in controlling the level of citrate and anthocyanin in postharvest TBO fruit in addition to PH4. These results may providing new insight into the regulation mechanism of citrate and anthocyanin accumulation in citrus fruit.

**Supplementary Information:**

The online version contains supplementary material available at 10.1186/s12870-023-04309-5.

## Background

Citrus is one of the most important fruit crops worldwide. China is the largest producer of citrus in the world with a current annual yield of 45 million tons, among which 90% are used for fresh consumption. With the changes in people’s consumption concept, ‘high quality’ has become the core competitiveness of fresh citrus fruit, and postharvest fresh-keeping technologies play an increasingly prominent role in citrus industry. Organic acids are important components for the flavor and quality of citrus fruit, and are closely related to the fruit storage performance [1, 2]. In addition, anthocyanin is an important component for citrus fruit color, as well as plays a critical role in citrus fruit quality formation, stress resistance and human health benefits [3–5]. Blood orange is the only commercial citrus variety that can accumulate anthocyanins in its fruit. The content of organic acid and anthocyanin jointly determine the quality of postharvest blood orange fruit.

The intracellular organic acid level is regulated by the synthesis, degradation, transport and vacuole storage of organic acids. A variety of enzymes involved in citrate metabolism have been reported, such as phosphoenolpyruvate carboxylase (PEPC) [6], phosphoenolpyruvate carboxylase kinase (PEPCK) [7], citrate synthase (CS) [8], cis-aconitase (Aco) [9], vacuolar proton pump V-ATPase/V-PPase and citrate/H^+^ cotransporter (Cit) [10, 12], GABA shunt gene and citrate lyase (ACL) [13, 14]. Moreover, transcription factors (TFs) including ERF, bHLH, NAC, WRKY, bZIP and MYB have also been reported to participate in the regulation of citrate level through the *VHA* [15], *Aco* [16, 17], *ACL* [18] or *PH8* [12, 19, 20] pathway.

The biosynthesis of anthocyanins is a complex multi-step process involving many structural and regulatory genes. These structural genes code enzymes like phenylalanine ammonia-lyase (PAL), chalcone synthase (CHS), chalcone isomerase (CHI), flavanone 3-hydroxylase (F3H), flavonoid 3’-hydroxylase (F3’H), dihydroflavonol 4-reductase (DFR), anthocyanidin synthase (ANS) and glutathione S transferase (GST) [21, 22]. The most extensively studied TFs for the transcriptional regulation of anthocyanin biosynthesis are MYB, bHLH and WD40 [23]. It has been demonstrated that MYB can regulate anthocyanin accumulation by directly binding to the promoters of key genes in the anthocyanin biosynthesis pathway [24, 25]. In general, bHLH needs to form a complex with the MYB protein to be involved in anthocyanin biosynthesis [4, 26–28]. Similarly, WD40 cannot independently regulate anthocyanin synthesis and mainly functions by forming MYB-bHLH-WD40 (MBW) complex [29]. It has been reported that anthocyanin accumulation in almost all citrus cultivars is closely associated with the activity of the *Ruby1* gene (a MYB TF) [30]. The *Ruby1* gene can respectively interact with bHLH and WD40 to regulate anthocyanin metabolism in citrus [4, 31].

Although numerous genes related to citrate and anthocyanin metabolism have been reported, little is known about the common regulatory pathways involved in the accumulation of both citrate and anthocyanins. In petunia, it has been reported that MYB complexes with bHLH and WD40 protein to regulate anthocyanin biosynthesis, and bHLH is actually the AN1 protein involved in regulating vacuolar acidification [32]. A recent study demonstrated that the traits of exceptionally low fruit acidity and absence of anthocyanins in leaves and flowers and proanthocyanidins in citrus seeds are due to the mutation of the *Noemi* gene encoding a bHLH TF (a homolog of AN1 in petunia and TT8 in *Arabidopsis*) [33]. Because both acidity and coloration are important fruit quality traits, increasing research has been focused on the discovery of new *Noemi* genes [34].

Transcriptome profiling has greatly contributed to the identification of new genes or pathways for many important traits [21, 35, 36]. Blood orange is the only commercial citrus variety that can accumulate anthocyanins in its fruit, and the acid content in its fruit is higher among sweet orange varieties. ‘Tarocco’ blood orange (TBO) is the main variety of blood orange grown in Hunan. ‘Bingtangcheng’ sweet orange (BTSO) is an excellent variety selected in Hunan province, and is characterized by a low acid content. It is also the main sweet orange variety planted in Hunan province. In this study, we comparatively analyzed the transcriptome of these two contrasting sweet orange germplasm, TBO and BTSO, throughout the postharvest storage process, aiming to identify the genes playing important roles in the postharvest process of citrus fruit, and thereby provide insights into the key pathways and regulators simultaneously associated with the accumulation of citrate and anthocyanins in citrus fruit.

## Results

### Variations in the physical and chemical characteristics of TBO and BTSO during postharvest storage

**Fig. 1:**
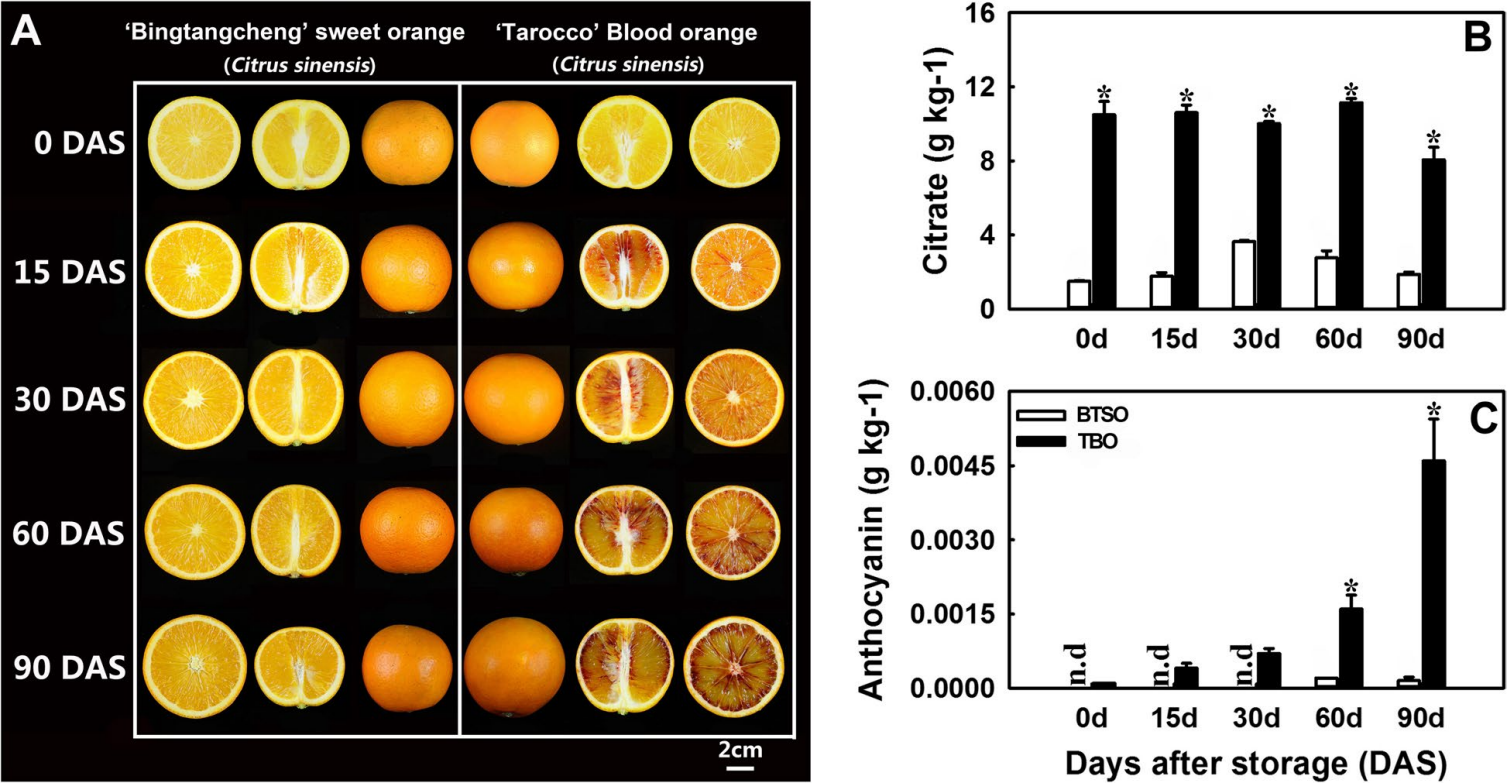
BTSO and TBO fruit under postharvest storage (**A**), and their corresponding citrate and anthocyanin contents (**B** and **C**). Error bars represent the standard deviations of the mean in three replicates, and for each storage day, * stand for significant differences between two materials at *p* < 0.05

To compare the fruit of TBO and BTSO, we examined the physiochemical characteristics of fruit at different stages of postharvest storage (Fig. 1). The two varieties exhibited similar fruit pulp colors at maturity (0 DAS, days after storage). Thereafter, the pulp of TBO fruit started to accumulate anthocyanins gradually from 15 to 90 DAS and was differentiated from BTSO fruit in color (Fig. 1A). Further determination of anthocyanin content revealed that it was gradually increased in TBO during storage and was significantly higher than that in BTSO (Fig. 1C).

Citrate and malate are major organic acids in citrus fruit. Hence, we also analyzed their changes in TBO and BTSO fruit during postharvest storage. The results showed that mature TBO (10.49 ± 0.72 g kg^−1^) had a markedly higher citrate content than BTSO (1.49 ± 0.05 g kg^−1^), which was consistently observed throughout the whole storage period (Fig. 1B). The malate content showed a decreasing tendency in TBO fruit, while a slightly increasing tendency in BTSO fruit along with storage (Fig. S1). In general, the malate content had little influence on total organic acids.

### Transcriptome analysis

**Fig. 2:**
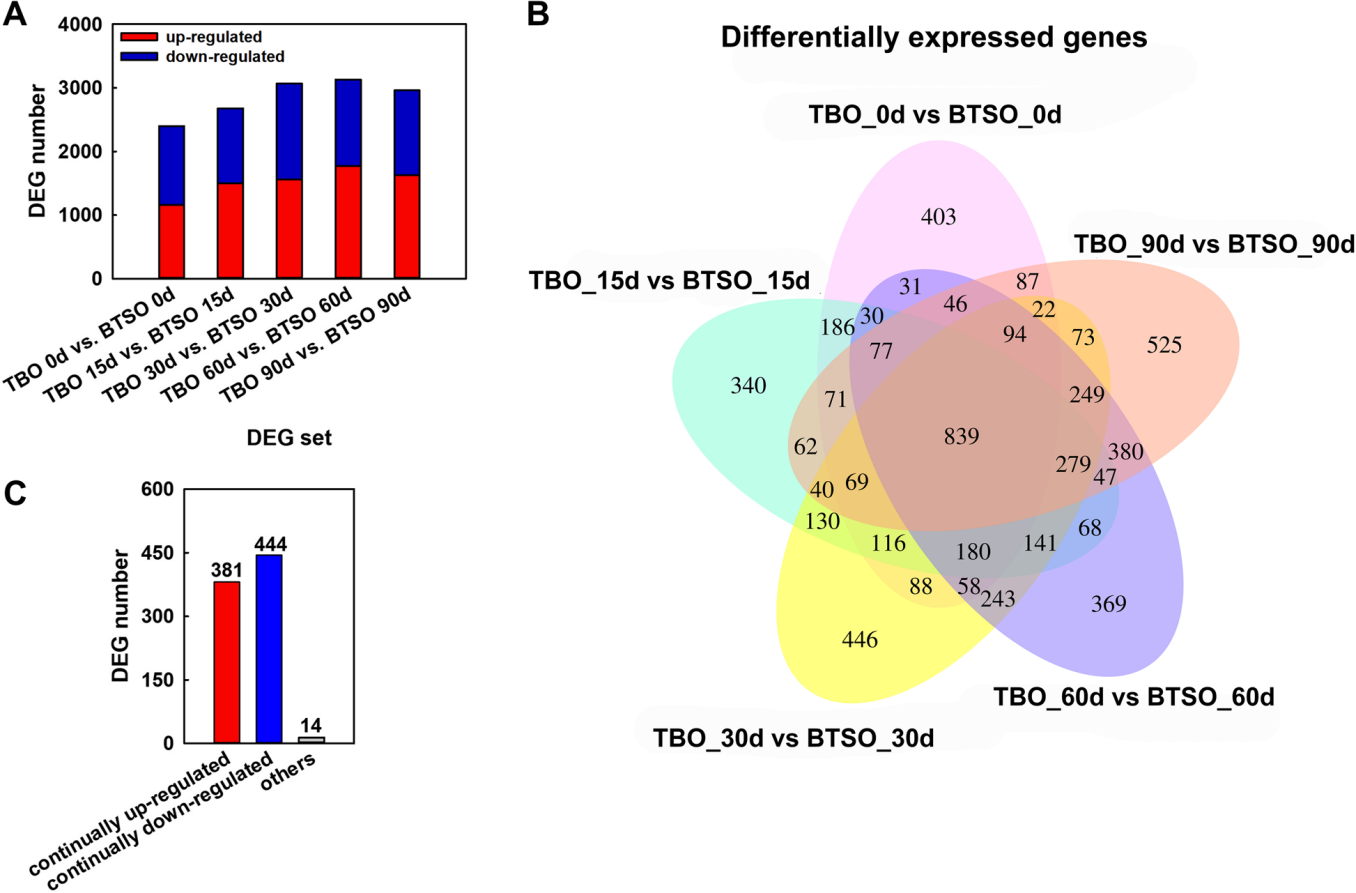
Summary of differentially expressed genes between TBO and BTSO fruit during postharvest storage. **A** Number of DEGs in different DEG sets. **B** Venn diagram shows DEGs in TBO vs BTSO. **C** Number statistics of DEGs intersected of TBO vs BTSO in five storage period groups

To explore the regulatory mechanism of citrate and anthocyanin accumulation in the flesh of citrus fruit, the samples at five detection time points (0, 15, 30, 60 90 DAS) from the two different varieties were used for deep RNA-seq analysis. After filtering out the rRNAs and low-quality reads, a total of 133 million reads were mapped to the *Citrus sinensis* reference genome (Table S2). For these clean reads, the average mapped reads per sample was greater than 90% (ranging from 87.90% to 91.87%). A total of 29,655 annotated genes were obtained. Differentially expressed genes (DEGs) were identified based on their expression levels in different samples, and functional annotation and enrichment analysis were performed. A total of 14,232 genes were differentially expressed between postharvest stored TBO and BTSO fruit (Fig. 2A). To screen the candidate genes related to citrate and anthocyanin biosynthesis, our study focused on the DEGs at TBO vs. BTSO. There were 2397, 2677, 3067, 3131 and 2960 genes that were differentially expressed on 0, 15, 30, 60 and 90 DAS respectively between TBO and BTSO (Fig. S2). Among them, 839 genes were consistently differentially expressed (Fig. 2B), and 381 genes were consistently upregulated, while 444 were consistently downregulated in TBO relative to BTSO throughout the storage period. The remaining 14 genes were upregulated in some storage periods and downregulated in other storage periods (Fig. 2C).

### Construction of a WGCNA and coexpression network

**Fig. 3:**
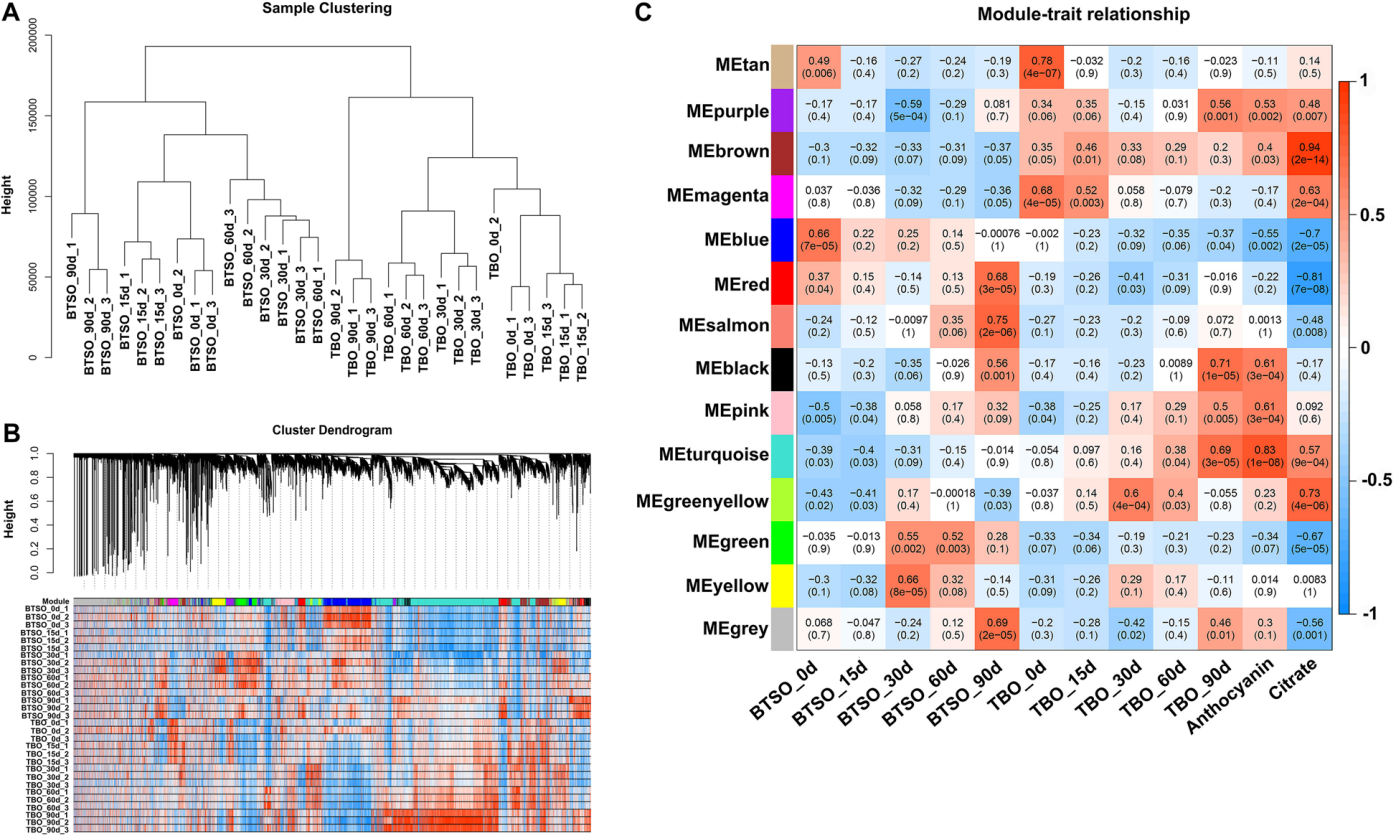
Weighted gene coexpression network analysis of TBO vs BTSO fruit during postharvest storage period. **A** Sample clustering. **B** Hierarchical clustering showing modules of coexpression genes. **C** Module/trait correlations and corresponding *p* values. The right panel shows a colour scale for module/trait correlations from -1 to 1

To obtain hub genes related to citrate and anthocyanin accumulation, the relationships of DEGs, citrate and anthocyanin content for each sample were analyzed by constructing a WGCNA (Fig. 3). Samples clustering showed that the three biological replicates of each sample were very good (Fig. 3A). Fourteen coexpression modules were identified by WGCNA (Fig. 3B), among which the turquoise module was highly positively correlated with the contents of citrate (*r* = 0.57, *p* value = 9e-04) and anthocyanin (*r* = 0.83, *p* value = 1e-08). In addition, the brown module was also highly positively correlated with the citrate content (*r* = 0.94, *p* value = 2e-14) (Fig. 3C). A total of 5154 and 1193 genes were obtained from turquoise and brown module respectively. Thinking about the content of citrate and anthocyanin were all markedly higher in TBO than that in BTSO fruit during the whole storage period (Fig. 1B and C), we focused on intersection genes between the 825 continually DEGs (Fig. 2C) and the turquoise and brown modules. Finally, 310 valuable genes was filtered out. According to GO and KEGG enrichment of the 310 candidate genes, 11 genes were mapped to the flavonoid metabolism pathway, 5 genes were mapped to the citrate cycle pathway, and 7 genes were mapped to the starch and sucrose metabolism pathway (Fig. S3 and S4).

### Identification of candidate genes involved in citrate and anthocyanin accumulation

**Fig. 4:**
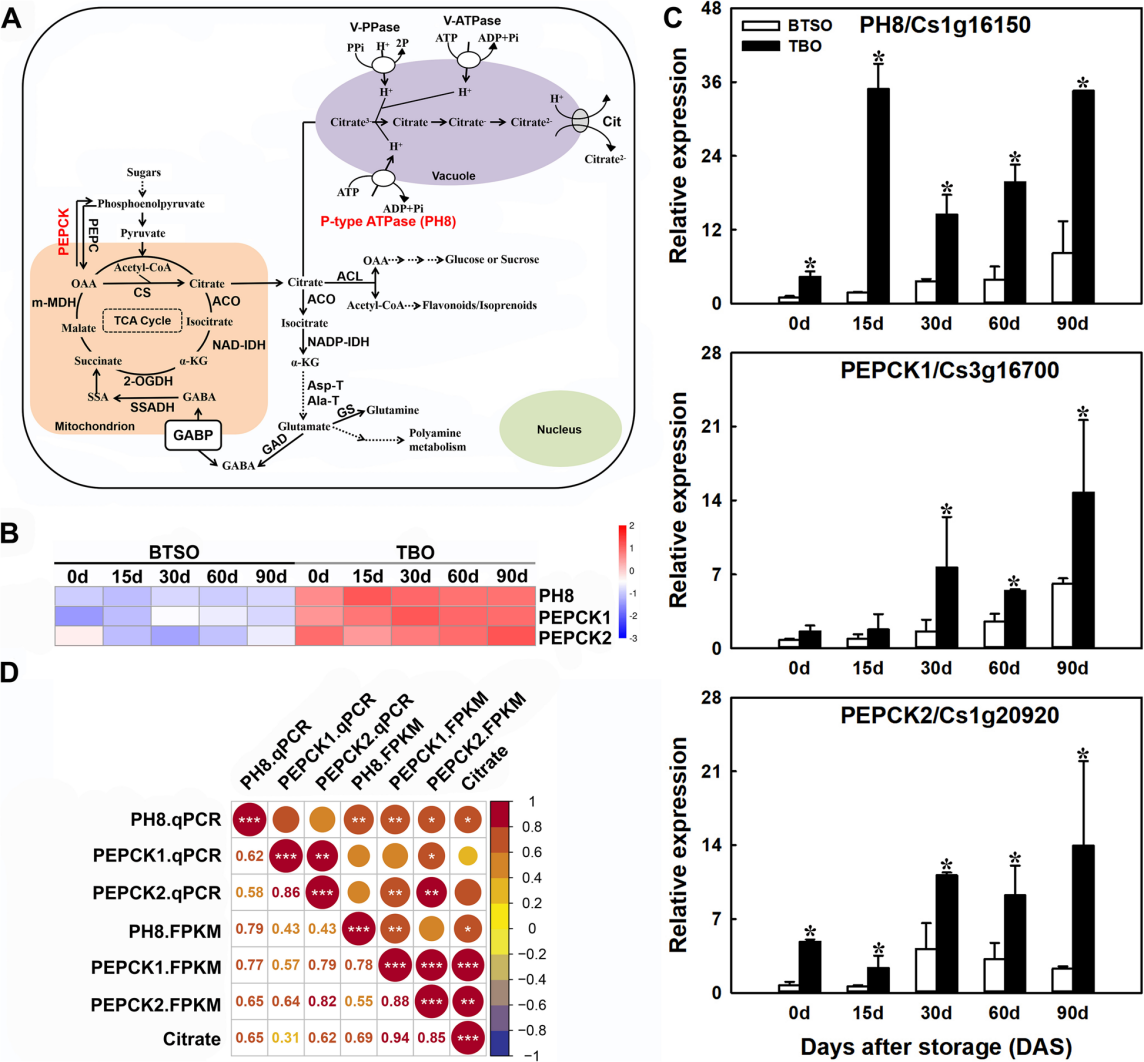
Analysis of genes related to citrate metabolism in the intersection genes between the 825 continually DEGs and the turquoise and brown modules. **A** Citrate metabolism pathway. The red font indicates the genes obtained in the intersection genes. **B** Heatmap of the expression levels of differentially expressed genes (DEGs) involved in citrate metabolism. **C** qRT-PCR detection of citrate metabolism related structural genes, *PH8* (Cs1g16150), *PEPCK1* (Cs3g16700) and *PEPCK2* (Cs1g20920). **D** Correlation analysis of the expression profiles in qRT-PCR (qPCR) and transcriptome data (FPKM) (*, ** and *** represent significant differences at *p* < 0.05, *p* < 0.01 and *p* < 0.001, respectively between the two sets of data)

**Fig. 5:**
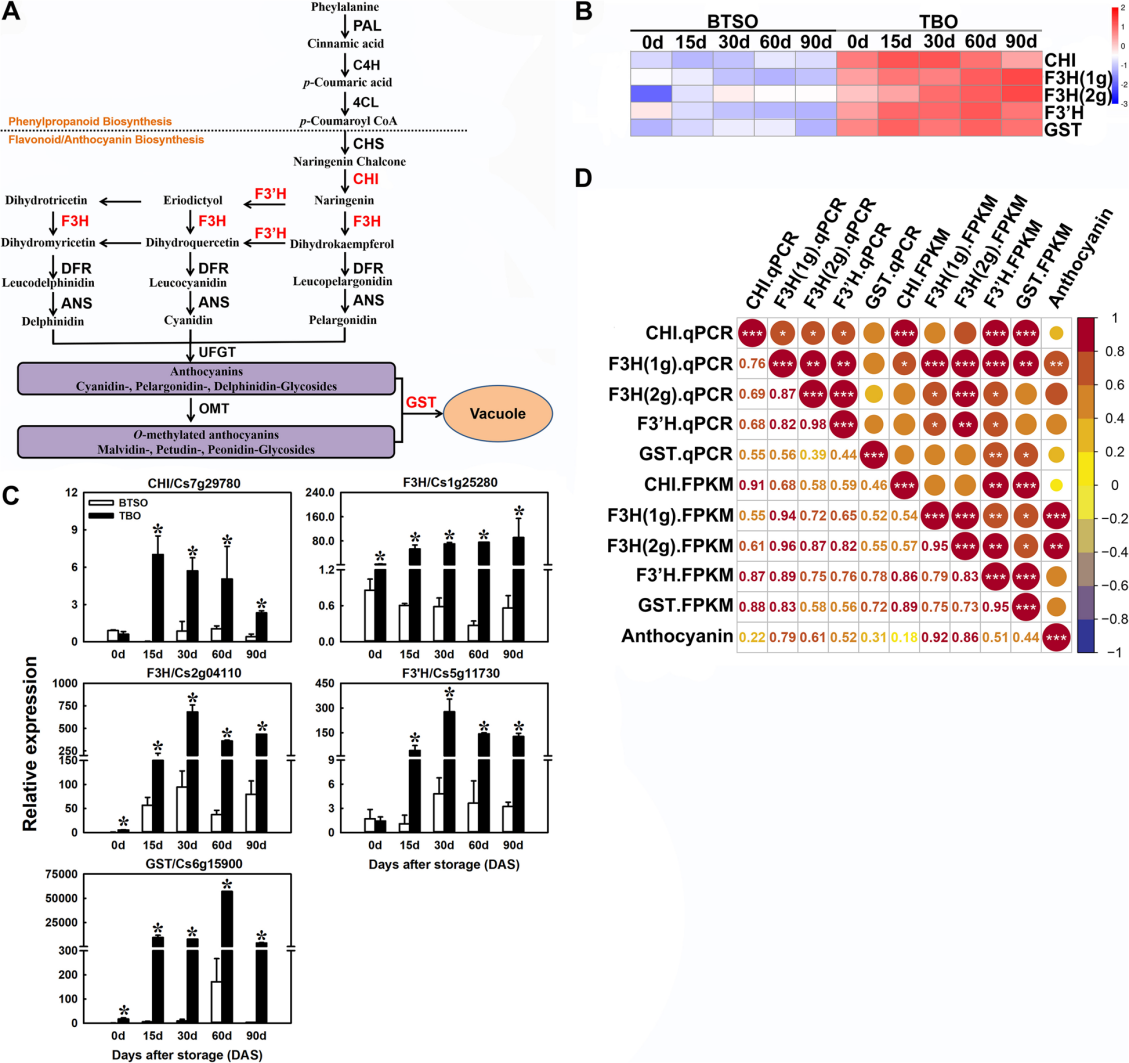
Analysis of genes related to anthocyanin biosynthesis in the intersection genes between the 825 continually DEGs and the turquoise and brown modules. **A** Anthocyanin biosynthesis pathway. The red font indicates the genes obtained in the intersection genes. **B** Heatmap of the expression levels of differentially expressed genes (DEGs) involved in anthocyanin biosynthesis. **C** qRT-PCR detection of anthocyanin biosynthesis related structural genes, *CHI* (Cs7g29780), *F3H* (Cs1g25280 and Cs2g04110), *F3’H* (Cs5g11730) and *GST* (Cs6g15900). **D** Correlation analysis of the expression profiles in qRT-PCR (qPCR) and transcriptome data (FPKM) (*, ** and *** represent significant differences at *p* < 0.05, *p* < 0.01 and *p* < 0.001, respectively between the two sets of data)

As a results, 3 and 5 structural genes involved in citrate metabolism and anthocyanin biosynthesis were obtained in 310 candidate genes. A heatmap of their expression profiles in the flesh of TBO and BTSO fruit was drawn based on their FPKM value (log2(FPKM)) (Figs. 4 and 5). The 3 structural genes from all major steps of the citrate metabolism pathway were distributed as follows: p-type ATPase gene (*PH8*), two phosphoenolpyruvate carboxylase kinase (*PEPCK*). Further the expression patterns of these 3 structural genes involved in citrate metabolism, *PH8* (Cs1g16150), *PEPCK1* (Cs3g16700) and *PEPCK2* (Cs1g20920), were studied via qRT-PCR (quantitative real-time PCR), and the transcripts of *PH8* and *PEPCK2* genes were significantly higher in TBO fruit than in BTSO fruit during the whole storage period (Fig. 4C), which was consistent with the results of transcriptome analysis based on the correlation analysis (Fig. 4D).

The 5 structural genes from all major steps of the anthocyanin biosynthesis pathway were distributed as follows: chalcone isomerase (*CHI*), two flavanone 3-hydroxylase (*F3H*), flavonoid 3’-hydroxylase (*F3’H*) and glutathione S transferase (*GST*). Further the expression patterns of these 5 structural genes involved in anthocyanin biosynthesis, *CHI* (Cs7g29780), *F3H* (Cs1g25280 and Cs2g04110), *F3’H* (Cs5g11730) and *GST* (Cs6g15900), were studied via qRT-PCR, and the transcripts of these genes were significantly higher in TBO fruit than in BTSO fruit during the whole storage period (Fig. 5C), which was consistent with the results of transcriptome analysis based on the correlation analysis (Fig. 5D).

### Identification of genes involved in carbohydrate metabolism

**Fig. 6:**
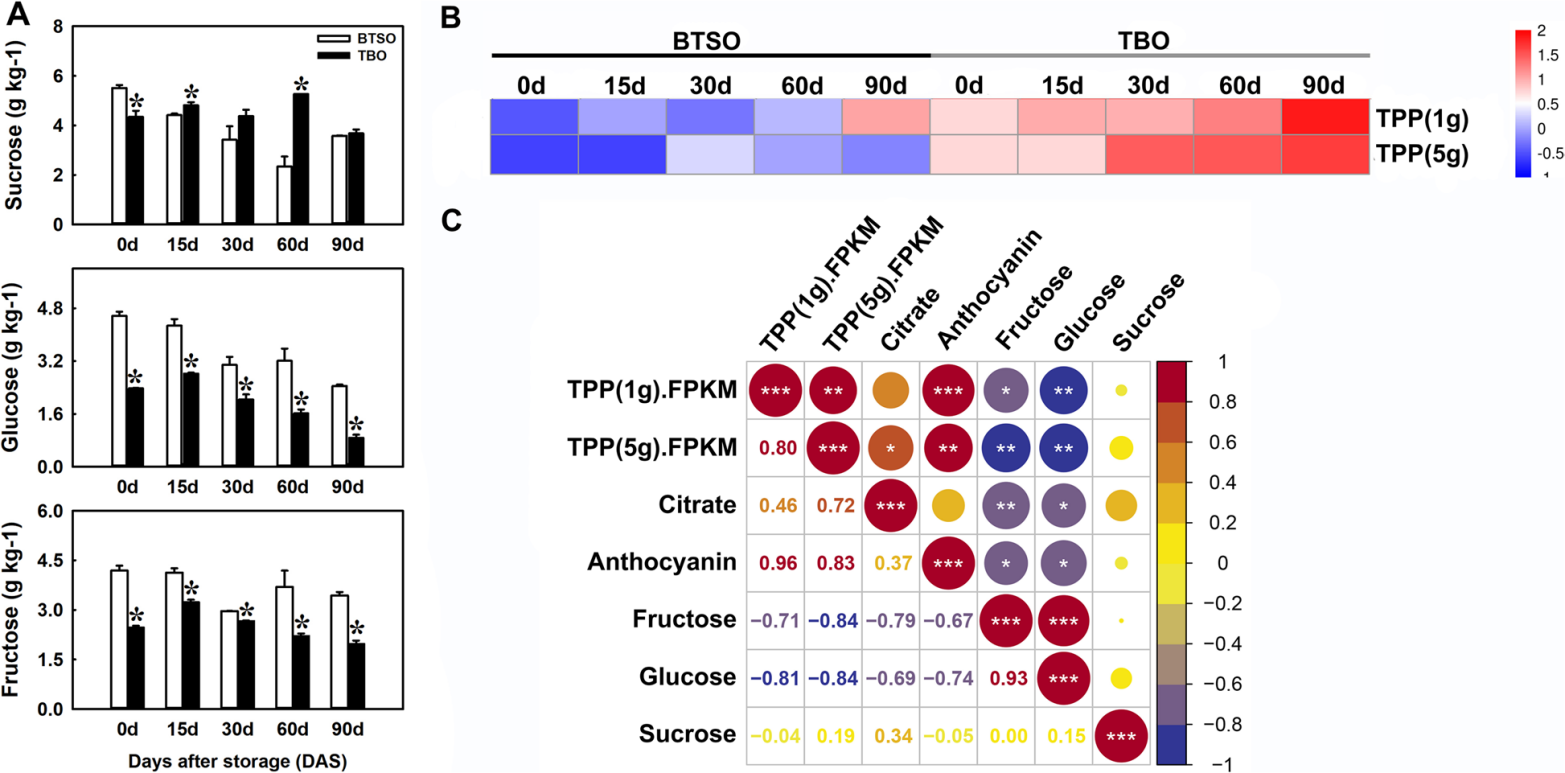
Soluble sugar content and expression pattern of carbohydrate metabolism related genes in TBO and BTSO fruit during postharvest storage period. **A** Glucose, fructose and sucrose contents in TBO fruit compared with BTSO fruit during storage. **B** Heatmap of the expression levels of carbohydrate metabolism related genes in the intersection genes between the 825 continually DEGs and the turquoise and brown modules. **C** Correlation analysis of the citrate content, anthocyanin content, soluble sugar content and expression profiles of carbohydrate metabolism related genes (*, ** and *** represent significant differences at *p* < 0.05, *p* < 0.01 and *p* < 0.001, respectively between the two sets of data). For each storage day, different lowercase letters stand for significant differences between two materials at *p* < 0.05

Carbohydrates are considered substrates for anthocyanin synthesis, and was also closely connect with organic acid metabolism. In our results, the contents of glucose, fructose and sucrose showed downward trends on the whole in TBO and BTSO fruit during postharvest storage. The glucose and fructose content in TBO fruit was significantly lower than that in BTSO throughout the storage period; while the sucrose content was markedly higher in TBO fruit than that in BTSO fruit at 15 and 60 DAS (Fig. 6A). In 310 candidate genes, two trehalose-phosphate phosphatase (*TPP*) genes were involved in starch and sugar metabolism which was related to citrate metabolism and anthocyanin synthesis (Fig. 6B). Correlation analysis showed that the expression patterns of the *TPP* genes were positively correlated with citrate and anthocyanin content, and was negatively correlated with glucose and fructose content (Fig. 6C), suggesting that higher expression levels of these genes were beneficial to carbohydrate metabolism, which contributed to citrate and anthocyanin accumulation in TBO fruit.

### Screening potential transcription factors that regulate citrate and anthocyanin accumulation in postharvest TBO fruit

**Fig. 7:**
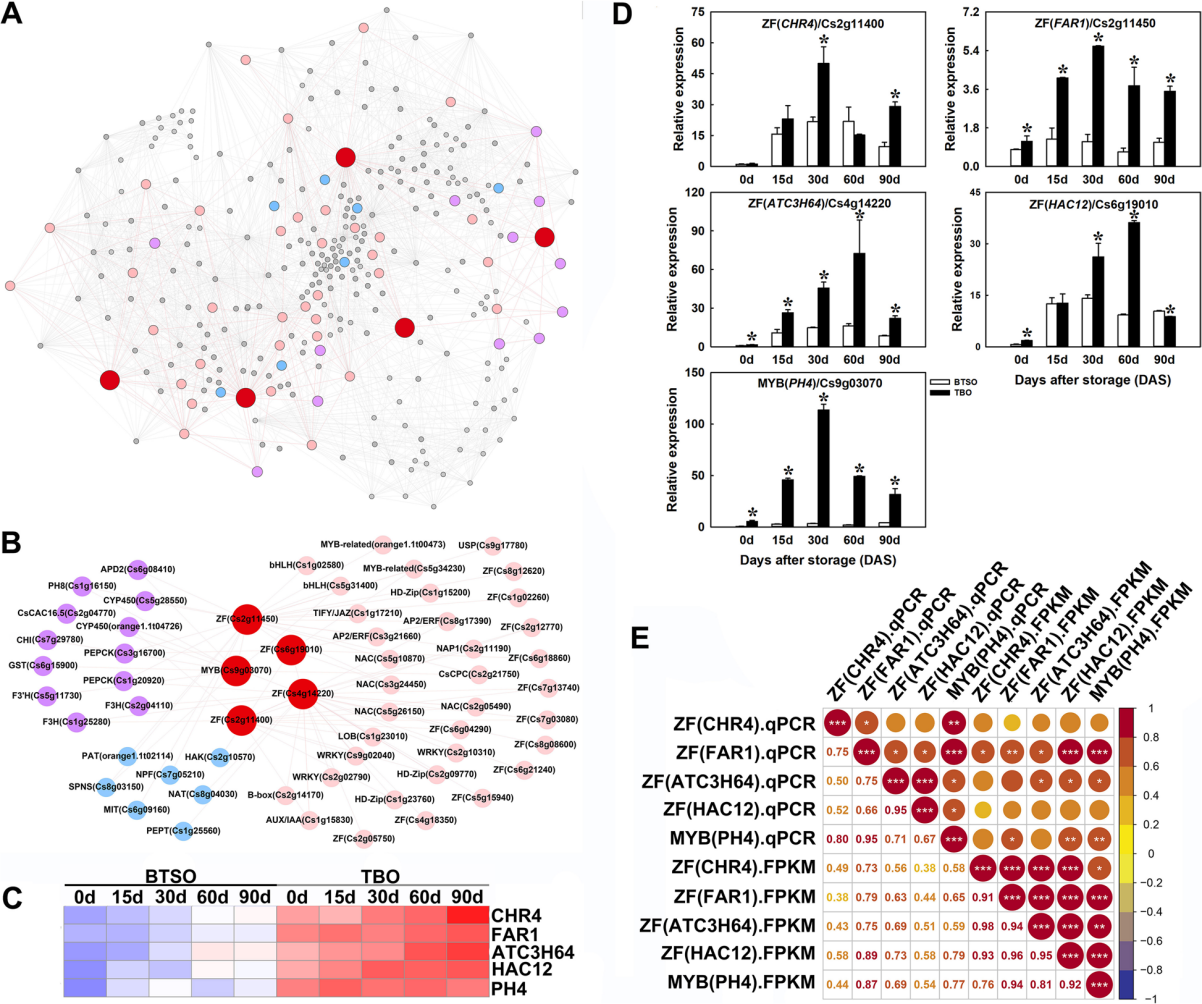
Coexpression network analysis of the potential key genes in the intersection between the 825 continually DEGs and the turquoise and brown modules. **A** coexpression network of the genes (weight > 0.15). **B** coexpression network of transcription factors and citrate/anthocyanin metabolism related structural genes. **C** Heatmap of the expression levels of the candidate transcription factors. **D** qRT-PCR detection of candidate transcription factor genes *CHR4* (Cs2g11400), *FAR1* (Cs2g11450), *ATC3H64* (Cs4g14220), *HAC12* (Cs6g19010) and *PH4* (Cs9g03070). **E** Correlation analysis of the expression profiles in qRT-PCR (qPCR) and transcriptome data (FPKM) (*, ** and *** represent significant differences at *p* < 0.05, *p* < 0.01 and *p* < 0.001, respectively between the two sets of data)

To explore the molecular regulatory mechanism of citrate metabolism and anthocyanin biosynthesis in TBO fruit, we constructed a coexpression network based on the genes present in the intersection of 825 continually differentially expressed genes (DEGs) (Fig. 2C) and turquoise + brown modules. In the network, we identified a total of 40 transcription factor genes, and 5 of them including PH4 (Cs9g03070), CHR4 (Cs2g11400), FAR1 (Cs2g11450), ATC3H64 (Cs4g14220), and HAC12 (Cs6g19010) were identified as hub genes (Fig. 7A and B). These TFs were found to be closely correlated with citrate and anthocyanin metabolism-related genes. Among them, PH4 was closely related to *PH8*, *CHI*, *F3’H*, and *GST*; CHR4 was closely related to *F3H* (Cs1g25280); FAR1 was closely related to *PH8*, *PEPCK1*, and *PEPCK2*; ATC3H64 was closely related to *PEPCK2* and *F3H* (Cs1g25280 and Cs2g04110); HAC12 was closely related to *PEPCK1* (Fig. 7B).

The heatmap based on the transcriptome data showed that the expression levels of *PH4*, *CHR4*, *HAC12*, *FAR1* and *ATC3H64* were obviously upregulated in the TBO fruit compared with BTSO fruit throughout the storage period (Fig. 7C). Meanwhile, the qRT-PCR results indicated that the transcripts of these 5 detected genes in the TBO fruit were obviously higher than those in BTSO fruit during storage, especially the *FAR1*, *ATC3H64* and *PH4* (Fig. 7D), which was consistent with the transcriptome analysis results based on the correlation analysis (Fig. 7E). Furthermore, the expression patterns of the 5 transcription factor genes were positively correlated with the changes of structural genes expression levels and citrate/anthocyanin content (Fig. S5). It was proposed that *PH4*, *CHR4*, *HAC12*, *FAR1* and *ATC3H64* might be involved in citrate and anthocyanin accumulation by regulating the expression of structural genes associated with citrate metabolism and anthocyanin biosynthesis, especially the *FAR1*, *ATC3H64* and *PH4* genes.

## Discussion

### The mechanism of higher content of citrate and anthocyanin in postharvest TBO fruit

Accumulations of citrate and anthocyanins are important indicators to evaluate citrus fruit quality, as well as important traits for consumers’ concern. Blood orange is the only commercial citrus variety that can accumulate anthocyanins in its fruit, and with higher organic acid content in its fruit compared with other sweet orange, which determined its unique flavor. Some studies have suggested that *PH8* might be responsible for the difference in acidity among mature or postharvest stored citrus varieties [1, 2, 7]. Here, the differentially expressed structural gene *PH8* was found based on the transcriptome data, and was mapped to the citrate metabolism pathway (Fig. 4A and B). Moreover, its expression pattern was significantly positively correlated with citrate content (Fig. 4 C and D), which was similar to previous study. These results indicate that in TBO fruit, more citrate accumulated in vacuoles due to the strikingly higher expression of *PH8*, which increases the ability of the proton pump to uptake citrate into vacuoles.

Additionally, a series of studies showed that the accumulation of anthocyanin in blood orange fruit was attributed to the upregulation of genes across the biosynthetic pathway [37, 38]. In this study, the differentially expressed structural gene *CHI, F3H, F3’H* and *GST* were found based on the transcriptome data, and was mapped to the anthocyanin biosynthesis pathway (Fig. 5A and B). The expression pattern of these genes was significantly positively correlated with anthocyanin content, especially two *F3H* genes (Fig. 5C and D). These results indicate that the high expression of *F3H* might be the main reason for anthocyanin accumulation during postharvest storage of TBO fruit, and the significant high expression of *F3’H* and *GST* also jointly promoted the anthocyanin accumulation.

### Transcription factors involved in citrate and anthocyanin accumulation in postharvest TBO fruit

The regulation of citrate metabolism has always been the concern of researchers. And so far, some transcription factors have been reported to be involved in the regulation of citrate metabolism through triggering structural genes [12, 33, 39–41]. MYB5a, MYB5b and MYBA1 have been proven to be involved in vacuolar acidification by inducing the expression of vacuolar acidification-related genes *PH5* and *CAC16.5* in grapevine [40]. The co-exprssion of *PH4* and *AN1* strongly induced *PH1* and *PH5* expression to lead to citrate accumulation in citrus fruit [12, 41]. *VvMYB5* has similar functions to *PhPH4* and *AtMYB5*, and is also involved in controlling vaculor hyper-acidification and trafficking in grapvine [39]. In the present study, both transcriptome analysis and qPCR quantitative detection that the expression of *PH4* was significantly higher in TBO fruit than that in BTSO, and was coordinated with the expression patterns of *PH8* (Fig. 7). Thus, the transcription factor PH4 is likely involved in regulating citrate accumulation in postharvest TBO fruit. Except for *PH4*, the expression patterns of *FAR1* was also found to be closely related to citrate accumulation and changes in the *PH8*, and moreover, the expression patterns of *FAR1* was similar to *PH4*, indicating that the transcription factors FAR1 was also involved in regulating citrate accumulation in postharvest TBO fruit. The proposed function of FAR is that it play crucial roles in controlling the growth and development of plants, and it also proved to involved in regulating light-induced myo-inositol biosynthesis and an alternative ascorbate biosynthetic pathway [42, 43]. However, the roles in regulating citrate accumulation in postharvest fruit are unclear and need to be studied further.

Previous studies have well documented that the transcription factors families such as the R2R3-MYB, bHLH and WD40-domain proteins primarily regulate anthocyanin biosynthesis via regulating the structural genes in the anthocyanin biosynthesis pathway [24, 28]. In grapevine, the MYB5b can involve in anthocyanin biosynthesis through upregulating a subset of anthocyanin structural genes [40]. Additionally, overexpression of *LcMYB5* increased anthocyanin biosynthesis in tobacco and petunia either by directly activating the expression of key structural genes such as *DFR* or by indirectly up regulating the expression of endogenous *bHLH1* [44]. Recently, research showed that PH4 could directly upregulating the expression of *Noemi* gene and involved in proanthocyanin biosynthesis through activating the expression of PA biosynthetic genes like *DFR*, *ANS*, *ANR*, *LAR* and *UFGT2* in citrus [45]. In this study, the expression of *PH4* was also coordinated with the expression patterns of *CHI*, *F3’H* and *GST* (Fig. 7). Thus, the transcription factor PH4 was also likely to be involved in regulating anthocyanin biosynthesis in postharvest TBO fruit. Moreover, the expression patterns of *CHR4*, *FAR1*, *ATC3H64* and *HAC12* were similar to *PH4*, indicating that these TFs were have similar functions to PH4. A *FAR1* gene, 16 and 15 members from *C3H* and *ZF* subfamilies was identified and differentially expressed in purple-fleshed compared with white-fleshed sweetpotato through transcriptome analysis [46], suggesting that these transcription factors involved in anthocyanin accumulation in sweetpotato. However, the regulation mechanism of these transcription factors in anthocyanin biosynthesis in postharvest fruit are unclear and need to be studied further.

**Fig. 8:**
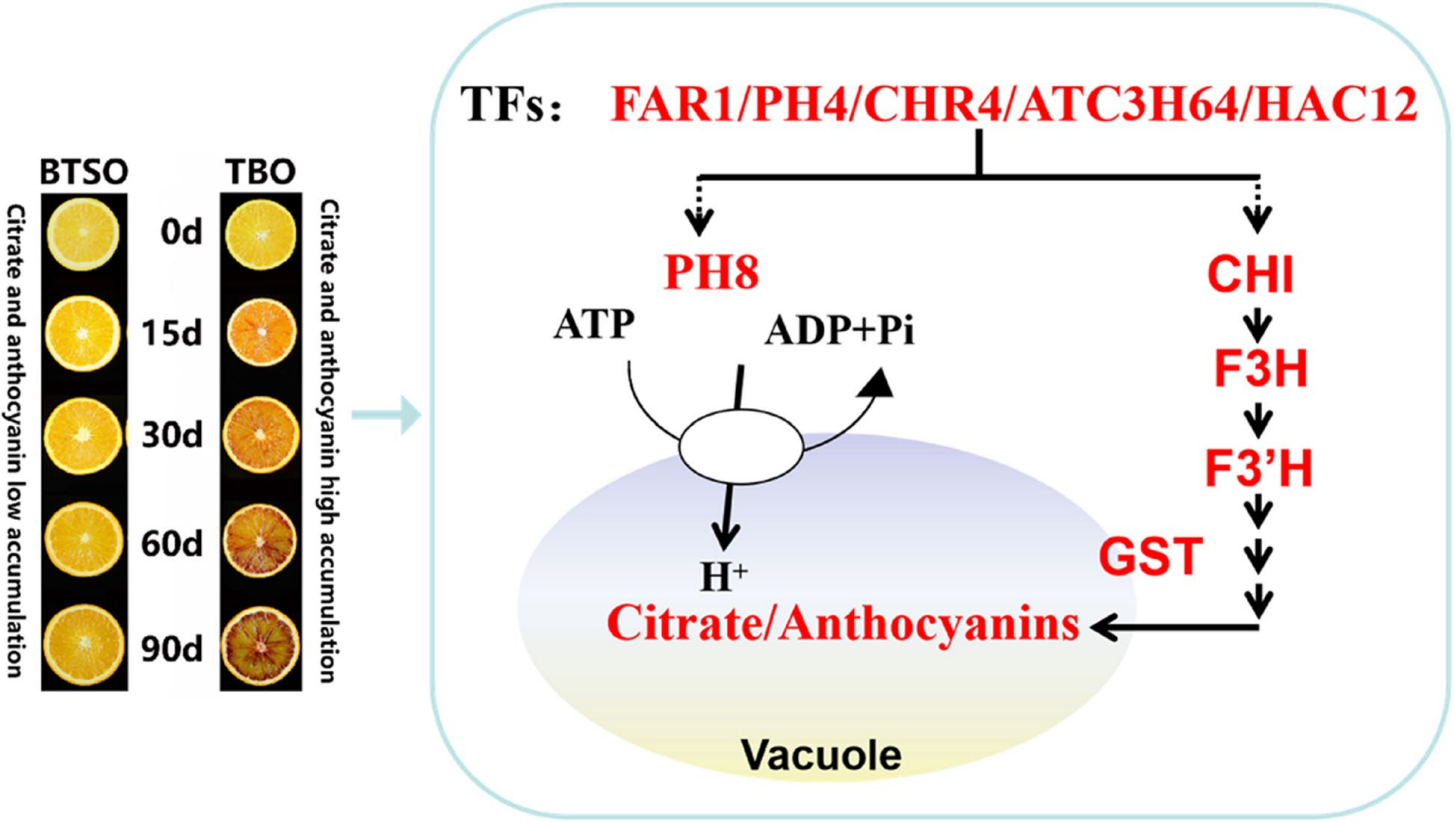
Sketch of the potential regulatory mechanisms involved in high citrate and anthocyanin trait in TBO fruits compared with that in BTSO fruits. The red typeface indicates that the metabolite content or gene expression level is significantly higher in TBO fruits than BTSO fruits

In recent years, many reports have proposed that the regulation of citrate metabolism is closely associated with that of anthocyanin biosynthesis [32, 33, 47]. The bHLH protein AN1 (also called Noemi) participates in the regulation of vacuolar acidification in petunia, and is also involved in the regulation of anthocyanin biosynthesis by complexing with MYB and WD40 protein [32]. The exceptionally low fruit acidity and the absence of anthocyanins in citrus leaves and flowers are due to mutation in the *Noemi* gene [33]. PH4 could directly upregulating the expression of *Noemi* gene and involved in both citrate accumulation and proanthocyanidin biosynthesis [45]. In this study, we found that the expression of *AN1* was also upregulated in TBO fruit and was significantly positive correlated with *PH4* (Fig. 7B). And so far, the transcription regulation involved in both citrate and anthocyanin metabolism are all around MYB and bHLH. Hence, with the results of this study, we hope to explore new regulatory factors from CHR4, FAR1, ATC3H64 and HAC12 that can simultaneously regulate the accumulation of citrate and anthocyanin. According to the results of previous studies [1, 4] and the present study, we established a model to explain the high citrate and anthocyanin accumulation in postharvest TBO fruits compared with BTSO fruits (Fig. 8).

## Conclusion

In this study, TBO and BTSO were chosen for a transcriptome analysis to excavate the key genes and regulators involved in citrate and anthocyanin metabolism in citrus fruit during postharvest storage. Importantly, we identified the key structural genes, transporters and TFs for the genetic control of citrate and anthocyanin accumulation in citrus fruit. Since in almost all transcriptome studies, it remains a great challenge to demonstrate which candidate genes play actual regulatory roles in fruit acidity and coloration, our comprehensive analysis provides an important basis for future study to determine whether the key genes identified here play important and specific roles in citrate and anthocyanin accumulation in citrus fruit.

## Materials and methods

### Plant materials

Physiologically mature fruit of ‘Tarocco’ blood orange (TBO) and ‘Bingtangcheng’ sweet orange (BTSO) (*Citrus sinensis*) were purchased from an experimental station in Huaihua, Hunan Province, China, in December 2019, and then immediately transported to the laboratory. Fruit with uniform size and color and free of any visible damage or defects were selected as samples for further experiments. The fruit were individually packed in polyethylene bags and stored in a temperature controlled chamber at 8 ± 1 °C with a relative humidity 85%–90% for about three months.

Juice sacs were separated from five fruit in each group at 0, 15, 30, 60 and 90 days after storage (DAS) with three replicates to measure the levels of organic acids, sugars, and for transcriptome analysis and expression analysis of related genes. The samples were immediately frozen in liquid nitrogen and stored at –80 °C for further analysis.

### Determination of organic acids and sugars

Organic acids (citrate and malate) and sugars (glucose, fructose and sucrose) were measured and analyzed by high-performance liquid chromatography (HPLC) with a previously described method [1]. The results were expressed on a fresh weight (FW) basis.

### Determination of anthocyanins

The anthocyanin contents were determined based on a previously reported protocol [48] with minor modifications. The mixed sample was ground in liquid nitrogen, and exactly 0.1 g of the sample was homogenized with 2 mL of A buffer (50 mM KCl and 150 mM HCl, pH = 1.0). Another 0.1 g of the sample was homogenized with 2 mL of B buffer (400 mM NaAC and 240 mM HCl, pH = 4.5). The homogenate was centrifuged at 12,000 rpm for 15 min at 4 °C. The supernatant was then collected and diluted to measure the A510 values, and the anthocyanin content (g kg^−1^ FW) = (A510 at pH1.0 – A510 at pH4.5) × 484.8 (molecular weight of cyanidin) / 24,825 (molar absorption coefficient of cyanidin at A510) × dilution ratio.

### Transcriptome sequencing and WGCNA 

Total RNA was extracted from TBO and BTSO fruit samples for 0, 15, 30, 60 and 90 DAS (three biological repetitions) using the Trizol reagent (Invitrogen, CA, USA) following the manufacturer's procedure. The mRNA was purified and fragmented into small pieces. Then, the cleaved RNA fragments were reverse transcribed to create the final cDNA library in accordance with the protocol for the mRNA Seq sample preparation kit (Illumina, San Diego, USA). RNA sequencing was performed on an Illumina Hiseq 4000 platform (LC-Bio, Hangzhou, China) to produce raw reads according to the protocol.

High-quality clean reads were used to de novo assemble the transcriptomes of TBO and BTSO using the StringTie program [49]. Then, all transcriptomes from samples were merged to reconstruct a comprehensive transcriptome using the Perl scripts. After the final transcriptome was generated, StringTie and Ballgown were used to estimate the expression levels of all genes. StringTie was used to determine the expression level for mRNAs by calculating the FPKM. Differentially expressed mRNAs and genes were selected with log_2_ (fold change) > 1 or log_2_ (fold change) < -1 and with statistical significance (*p* value < 0.05) by R package -Ballgown [50]. Gene Ontology (GO) annotation was performed using the eggNOG-mapper (http://eggnog-mapper.embl.de/). DEGs were enriched in GO and Kyoto Encyclopedia of Genes and Genomes (KEGG) databases so as to identify the changes in biological functions and metabolism pathways [51, 53]. Finally, the transcriptome data and phenotype data were analyzed by WGCNA using R language. The WGCNA package was employed to construct a gene coexpression network using a variant set of genes (15,318 genes, the maximum FPKM in all samples ≥ 2). The analysis was performed based on the WGCNA package in R studio software [54]. The main parameters of WGCNA program were as follows: variance data expression > 0,no mission data expression < 0.1; soft threshold = 18 (scale-free R2 = 0.9); deep split = 2; min module size = 60; merge cut height = 0.25.

### Quantitative real-time PCR validation

The candidate DEGs were verified by qRT-PCR. Total RNA from samples was extracted by the CTAB method and used for quantitative real-time PCR.

The RNA samples were used for first-strand cDNA synthesis using a FastKing gDNA Dispelling RT SuperMix reverse transcriptase Kit (Tiangen, China) following the manufacturer’s instructions. qRT-PCR assays were performed according to previous reports [1], and the relative expression levels were calculated with the 2^−ΔΔCt^ method in three biological replications. Specific primers were designed from the selected gene sequences using Primer Express 3.0 (Applied Biosystems, Foster City, CA, USA) and the primer sequences are given in Table S1.

### Statistical analysis

Each experiment was performed in three replicates. Experimental results were analyzed using IBM SPSS Statistics, RStudio and Gephi software. Error bars denote standard deviations. Different lowercase letters above the bars indicate significant differences at *p* < 0.05, which were obtained based on one-way ANOVA using IBM SPSS Statistics software.

## Supplementary Information

**Additional file 1: Table S1.** Primers used for quantitative real-time PCR analysis. **Table S2.** Number of reads after filtering rRNA and low quality. **Fig. S1.** The content of malate in TBO and BTSO fruit during postharvest storage. Error bars represent the standard deviations of the mean in three replicates, and for each storage day, different lowercase letters stand for significant differences between two materials at *p* < 0.05. **Fig. S2.** Volcano plots of differentially expressed genes (DEGs) in the comparisons of (A) TBO 0d vs. BTSO 0d, (C) TBO 15d vs. BTSO 15d, (E) TBO 30d vs. BTSO 30d, (G) TBO 60d vs. BTSO 60d, and (I) TBO 90d vs. BTSO 90d. Scatter plot of the KEGG pathway enrichment of DEGs. Rich factor is the ratio of the DEG number to the background number in a certain pathway. The size of the dots represents the number of genes, and the color of the dots represents the range of the q-value. B, D, F, H, and J present the top 20 terms at 0, 15, 30, 60 and 90 DAS between TBO and BTSO, respectively. **Fig. S3.** KEGG enrichment of the 310 valuable genes. **Fig. S4.** GO enrichment of the 310 valuable genes. **Fig. S5.** Correlation analysis of expression profiles of citrate metabolism and anthocyanin biosynthesis related genes, citrate and anthocyanin content. *, ** and *** represent significant differences at* p* < 0.05, *p* < 0.01 and *p* < 0.001, respectively.

## Data Availability

The data discussed in this publication have been deposited in NCBI's Gene Expression Omnibus and are accessible through GEO Series accession number GSE212015 https://www.ncbi.nlm.nih.gov/geo/query/acc.cgi?acc=GSE212015.

## Abbreviations

TBO: ‘Tarocco’ blood orange
BTSO: ‘Bingtangcheng’ sweet orange
WGCNA: Weighted gene coexpression correlation network analysis
PEPC: Phosphoenolpyruvate carboxylase
PEPCK: Phosphoenolpyruvate carboxylase kinase
CS: Citrate synthase
Aco: Cis-aconitase
Cit: Citrate/H^+^ cotransporter
ACL: Citrate lyase
TF: Transcription factors
PAL: Phenylalanine ammonia-lyase
CHS: Chalcone synthase
CHI: Chalcone isomerase
F3H: Flavanone 3-hydroxylase
F3’H: Flavonoid 3’-hydroxylase
DFR: Dihydroflavonol 4-reductase
ANS: Anthocyanidin synthase
GST: Glutathione S transferase
MBW: MYB-bHLH-WD40
PH8: P-type ATPase gene
TPP: Trehalose-phosphate phosphatase
