# Supplementary material for: Weighted gene coexpression correlation network analysis reveals the potential molecular regulatory mechanism of citrate and anthocyanin accumulation between postharvest ‘Bingtangcheng’ and ‘Tarocco’ blood orange fruit
Source: BMC Plant Biol. 2023 Jun 2;23:296. doi: 10.1186/s12870-023-04309-5 (PMC10236656; doi:10.1186/s12870-023-04309-5)
Supplement: Supplementary file 1 — Additional file 1: Table S1. Primers used for quantitative real-time PCR analysis. Table S2. Number of reads after filtering rRNA and low quality. Fig. S1. The content of malate in TBO and BTSO fruit during postharvest storage. Error bars represent the standard deviations of the mean in three replicates, and for each storage day, different lowercase letters stand for significant differences between two materials at p < 0.05. Fig. S2. Volcano plots of differentially expressed genes (DEGs) in the comparisons of (A) TBO 0d vs. BTSO 0d, (C) TBO 15d vs. BTSO 15d, (E) TBO 30d vs. BTSO 30d, (G) TBO 60d vs. BTSO 60d, and (I) TBO 90d vs. BTSO 90d. Scatter plot of the KEGG pathway enrichment of DEGs. Rich factor is the ratio of the DEG number to the background number in a certain pathway. The size of the dots represents the number of genes, and the color of the dots represents the range of the q-value. B, D, F, H, and J present the top 20 terms at 0, 15, 30, 60 and 90 DAS between TBO and BTSO, respectively. Fig. S3. KEGG enrichment of the 310 valuable genes. Fig. S4. GO enrichment of the 310 valuable genes. Fig. S5. Correlation analysis of expression profiles of citrate metabolism and anthocyanin biosynthesis related genes, citrate and anthocyanin content. *, ** and *** represent significant differences at p < 0.05, p < 0.01 and p < 0.001, respectively. [file 12870_2023_4309_MOESM1_ESM.doc]

Supplementary Material

**Weighted gene coexpression correlation network analysis reveals the potential molecular regulatory mechanism of citrate and anthocyanin accumulation between postharvest ‘Bingtangcheng’ and ‘Tarocco’ blood orange fruit**

**Yan Jin^1^,** ^#^**, Manyu Liao^1^,** ^#^**, Na Li^1^, Xiaoqian Ma^1^, Huimin Zhang^1^, Jian Han^2^, Dazhi Li^1^, Junfeng Yang^1^, Xiaopeng Lu^1^, Guiyou Long^1^, Ziniu Deng^1^, Ling Sheng^1^,***

^1^National Center for Citrus Improvement Changsha, College of Horticulture, Hunan Agricultural University, Changsha, CS, China

^2^Hunan Horticultural Research Institute, Changsha, CS, China

*Corresponding author.

E-mail: [shengling0629@163.com](mailto:shengling0629@163.com), Tel.: +86 073184638214, Fax: +86 073184638214)

^#^These authors contributed equally to this work.

Table S1. Primers used for quantitative real-time PCR analysis

| Gene Name | Gene ID | Primer 5’-3’ | |
| --- | --- | --- | --- |
| PH8 | Cs1g16150 | CCGTGAAGGAAGGAATGATTTGG | CCATGACAATGGATTCCACA |
| PEPCK1 | Cs3g16700 | GCCGACCAAGTTCTCAGACA | CACGCTAACATTCATCACCATC |
| PEPCK2 | Cs1g20920 | GGCTAGCGAAGATTCAAACG | GTCCCTTTAATGGGGGTGTT |
| CHIL | Cs7g20780 | GATGCTCTTGTCTCTGCTCCA | GTCCCTCACCGCACTTTCTA |
| F3H | Cs2g04110 | TCGAATGGAGGAACCCATCAC | CCATGGTTCACCAACTGAAAGA |
| F3H | Cs1g25280 | CCGGGAACCATCACACTCTT | CTCCCATTGCTCAGATAATGACC |
| F3’H | Cs5g11730 | CACGTTCGCCAGGAAGAGAT | CAACGCACAAGTTCAACCGT |
| GST | Cs6g15900 | CTTCTTCGACAGCCGTTTGG | GTTTGGCCCTTGGTTTGCAT |
| MYB | Cs9g03070 | CGAGCTTCTGGCCAACTACA | GAGAAGGCGATGTAGGCGAA |
| CHR4 | Cs2g11400 | ATCTGCGACGGCTACAGATG | AGGCGAAAGCTCAGCAAGAT |
| FAR1 | Cs2g11450 | AAGGGCTGGGACAACTGAAC | TGCAACTGAGAGGGTCCAAC |
| ATC3H64 | Cs4g14220 | AACTATGTGGAAGGCCGCTC | ATCGGTGACTTTGAAGCCGT |
| HAC12 | Cs6g19010 | CCAGCTGTGTCAAGCAGAGA | AACAATGCCGCATGCCATTT |

Table S2. Number of reads after filtering rRNA and low quality

| Sample | Total | Unmapped (%) | Unique_Mapped (%) | Multiple_Mapped (%) | Total_Mapped (%) | Genes (%) |
| --- | --- | --- | --- | --- | --- | --- |
| TBO 0d-1 | 46188252 | 3924563 (8.50%) | 33286610 (72.07%) | 8977079 (19.44%) | 42263689 (91.51%) | 19241 (64.88%) |
| TBO 0d-2 | 45669764 | 4129983 (9.04%) | 34074114 (74.61%) | 7465667 (16.35%) | 41539781 (90.96%) | 19336 (65.20%) |
| TBO 0d-3 | 44788832 | 3828892 (8.55%) | 32205841 (71.91%) | 8754099 (19.55%) | 40959940 (91.46%) | 19322 (65.16%) |
| TBO 15d-1 | 43109930 | 3833624 (8.89%) | 32021604 (74.28%) | 7254702 (16.83%) | 39276306 (91.11%) | 19411 (65.46%) |
| TBO 15d-2 | 45223532 | 4060921 (8.98%) | 33128652 (73.26%) | 8033959 (17.76%) | 41162611 (91.02%) | 19480 (65.69%) |
| TBO 15d-3 | 43386368 | 4127804 (9.51%) | 31614096 (72.87%) | 7644468 (17.62%) | 39258564 (90.49%) | 19624 (66.17%) |
| TBO 30d-1 | 47988548 | 5071791 (10.57%) | 34688926 (72.29%) | 8227831 (17.15%) | 42916757 (89.44%) | 20123 (67.86%) |
| TBO 30d-2 | 34994276 | 3169801 (9.06%) | 25595578 (73.14%) | 6228897 (17.80%) | 31824475 (90.94%) | 19582 (66.03%) |
| TBO 30d-3 | 42029492 | 4054379 (9.65%) | 30041458 (71.48%) | 7933655 (18.88%) | 37975113 (90.36%) | 19825 (66.85%) |
| TBO 60d-1 | 48618464 | 4204167 (8.65%) | 35059885 (72.11%) | 9354412 (19.24%) | 44414297 (91.35%) | 20541 (69.27%) |
| TBO 60d-2 | 48497198 | 4960805 (10.23%) | 34933170 (72.03%) | 8603223 (17.74%) | 43536393 (89.77%) | 20326 (68.54%) |
| TBO 60d-3 | 48363482 | 4728329 (9.78%) | 35150071 (72.68%) | 8485082 (17.54%) | 43635153 (90.22%) | 20498 (69.12%) |
| TBO 90d-1 | 50277832 | 6086787 (12.11%) | 35705954 (71.02%) | 8485091 (16.88%) | 44191045 (87.90%) | 20597 (69.46%) |
| TBO 90d-2 | 44153616 | 5232072 (11.85%) | 31854413 (72.14%) | 7067131 (16.01%) | 38921544 (88.15%) | 20218 (68.18%) |
| TBO 90d-3 | 43803078 | 4933662 (11.26%) | 31933142 (72.90%) | 6936274 (15.84%) | 38869416 (88.74%) | 20415 (68.84%) |
| BTSO 0d-1 | 44131880 | 3586451 (8.13%) | 31249132 (70.81%) | 9296297 (21.06%) | 40545429 (91.87%) | 19275 (65.00%) |
| BTSO 0d-2 | 46815470 | 4032534 (8.61%) | 33967127 (72.56%) | 8815809 (18.83%) | 42782936 (91.39%) | 19694 (66.41%) |
| BTSO 0d-3 | 45285968 | 3716390 (8.21%) | 31827574 (70.28%) | 9742004 (21.51%) | 41569578 (91.79%) | 19333 (65.19%) |
| BTSO 15d-1 | 44854442 | 3752252 (8.37%) | 31874523 (71.06%) | 9227667 (20.57%) | 41102190 (91.63%) | 19417 (65.48%) |
| BTSO 15d-2 | 36883434 | 3191840 (8.65%) | 26194260 (71.02%) | 7497334 (20.33%) | 33691594 (91.35%) | 19165 (64.63%) |
| BTSO 15d-3 | 42872158 | 3511542 (8.19%) | 29497791 (68.80%) | 9862825 (23.01%) | 39360616 (91.81%) | 19469 (65.65%) |
| BTSO 30d-1 | 47544246 | 4149751 (8.73%) | 32443372 (68.24%) | 10951123 (23.03%) | 43394495 (91.27%) | 19946 (67.26%) |
| BTSO 30d-2 | 51095942 | 4265474 (8.35%) | 36544893 (71.52%) | 10285575 (20.13%) | 46830468 (91.65%) | 20098 (67.77%) |
| BTSO 30d-3 | 46570278 | 4077745 (8.76%) | 33736186 (72.44%) | 8756347(18.80%) | 42492533 (91.24%) | 20058 (67.64%) |
| BTSO 60d-1 | 44179896 | 4634970 (10.49%) | 30703854 (69.50%) | 8841072(20.01%) | 39544926 (89.51%) | 19876 (67.02%) |
| BTSO 60d-2 | 43027960 | 3754160 (8.72%) | 31742851 (73.77%) | 7530949(17.50%) | 39273800 (91.27%) | 19706 (66.45%) |
| BTSO 60d-3 | 43739434 | 3877967 (8.87%) | 31437348 (71.87%) | 8424119(19.26%) | 39861467 (91.13%) | 19850 (66.94%) |
| BTSO 90d-1 | 37367180 | 3332632 (8.92%) | 27412044 (73.36%) | 6622504(17.72%) | 34034548 (91.08%) | 19943 (67.25%) |
| BTSO 90d-2 | 41269508 | 3655400 (8.86%) | 29771041 (72.14%) | 7843067(19.00%) | 37614108 (91.14%) | 19906 (67.13%) |
| BTSO 90d-3 | 45477404 | 3936239 (8.66%) | 33335815 (73.30%) | 8205350(18.04%) | 41541165 (91.34%) | 19969 (67.34%) |


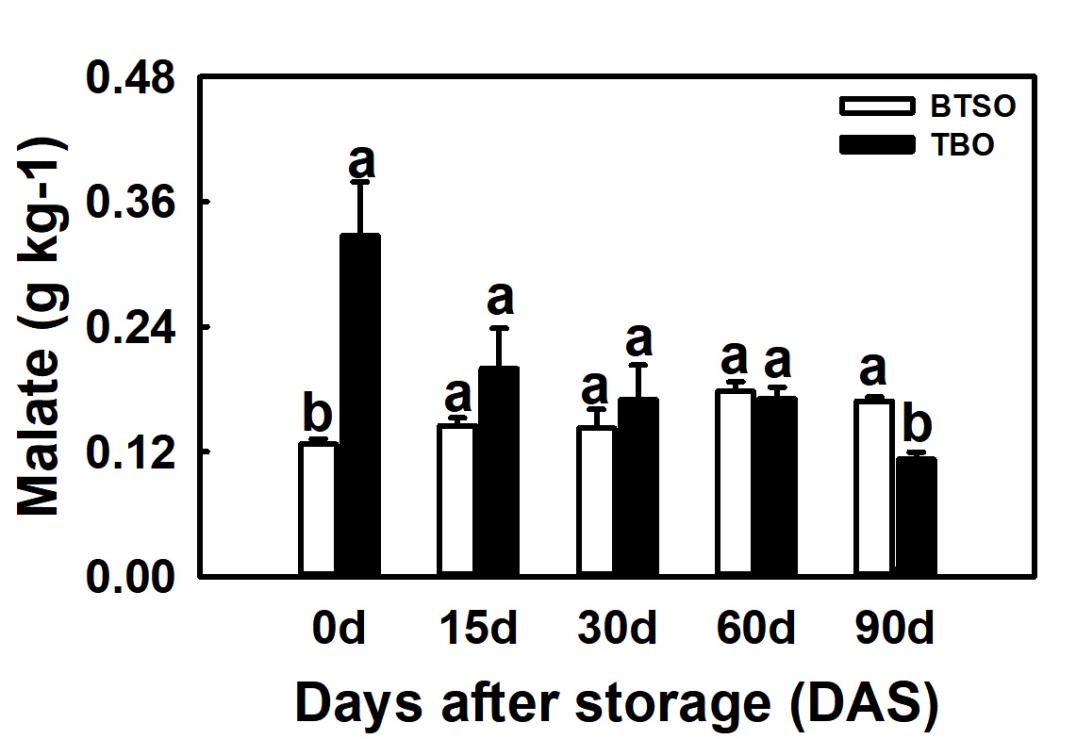


Fig. S1 The content of malate in TBO and BTSO fruit during postharvest storage. Error bars represent the standard deviations of the mean in three replicates, and for each storage day, different lowercase letters stand for significant differences between two materials at *p* < 0.05.


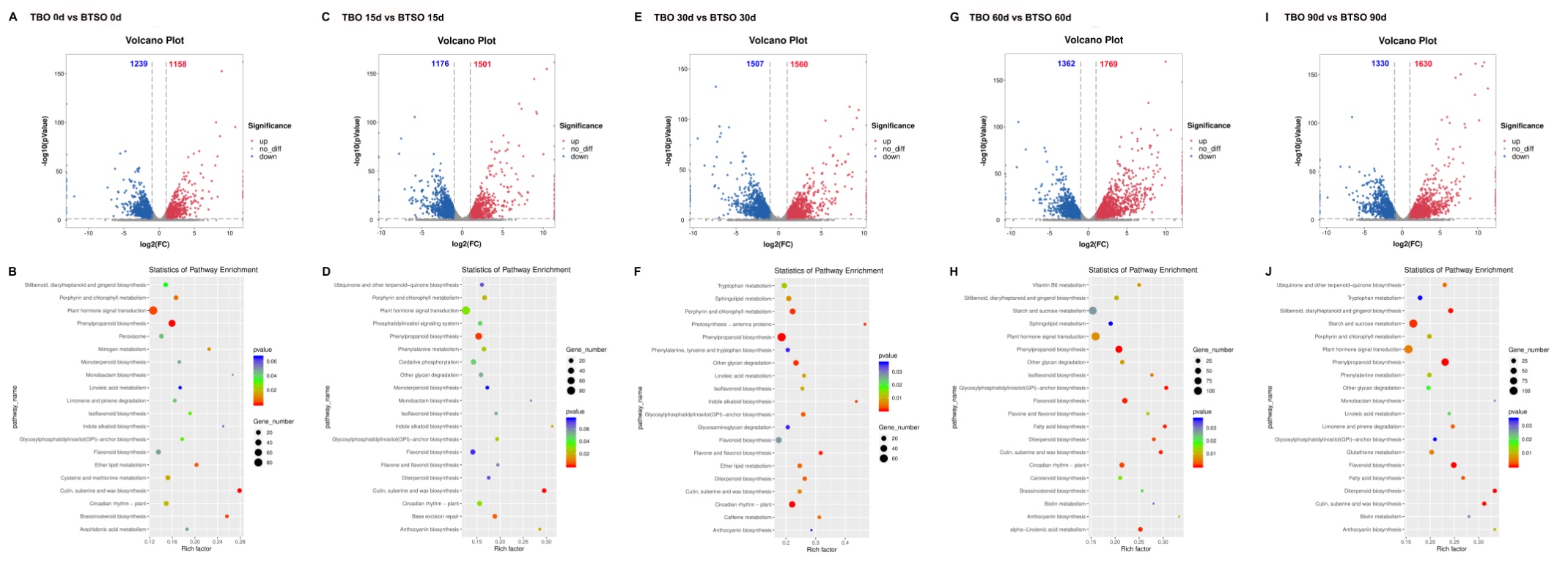


Fig. S2 Volcano plots of differentially expressed genes (DEGs) in the comparisons of (A) TBO 0d vs. BTSO 0d, (C) TBO 15d vs. BTSO 15d, (E) TBO 30d vs. BTSO 30d, (G) TBO 60d vs. BTSO 60d, and (I) TBO 90d vs. BTSO 90d. Scatter plot of the KEGG pathway enrichment of DEGs. Rich factor is the ratio of the DEG number to the background number in a certain pathway. The size of the dots represents the number of genes, and the color of the dots represents the range of the q-value. B, D, F, H, and J present the top 20 terms at 0, 15, 30, 60 and 90 DAS between TBO and BTSO, respectively.


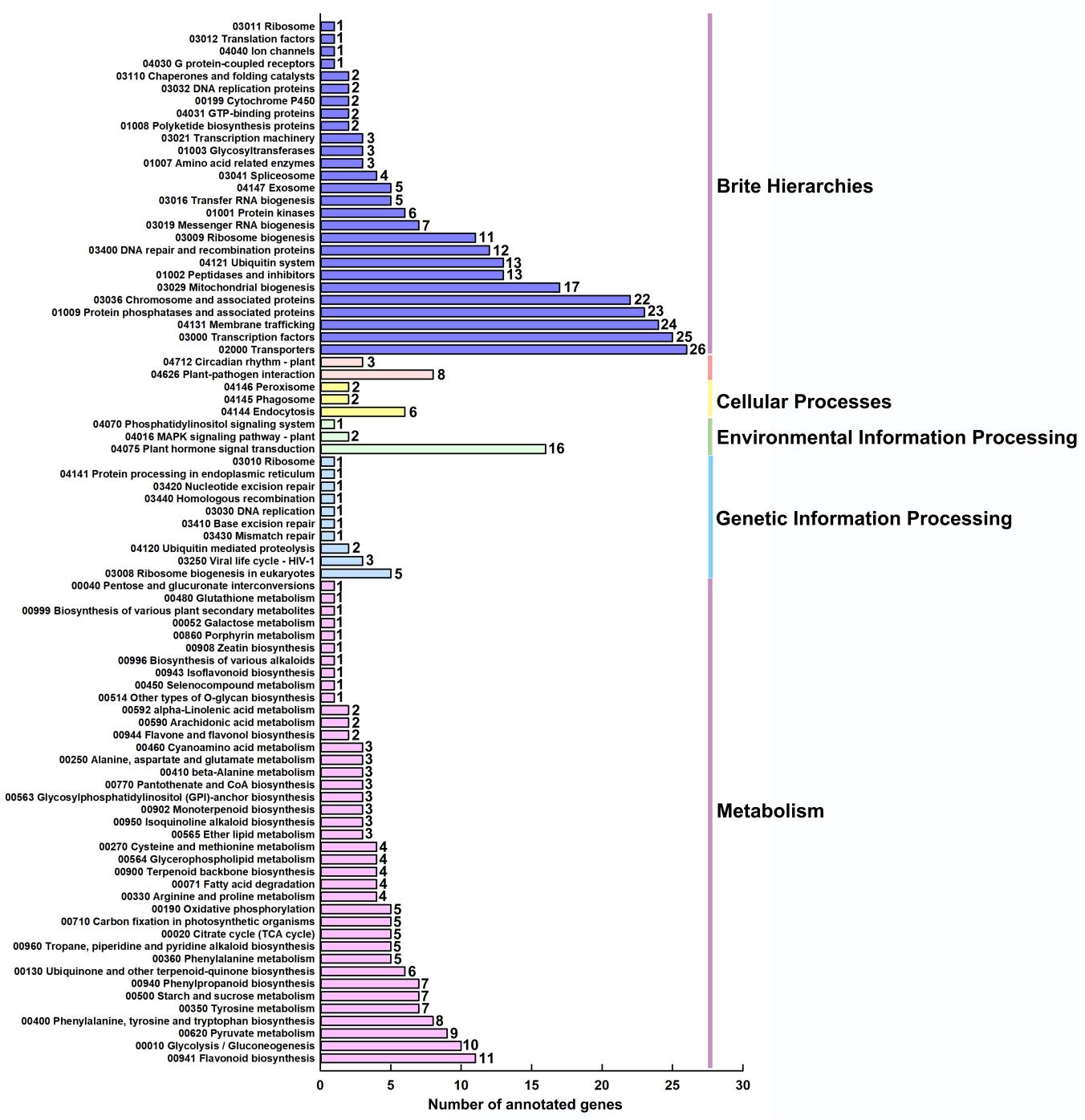


Fig. S3 KEGG enrichment of the 310 valuable genes


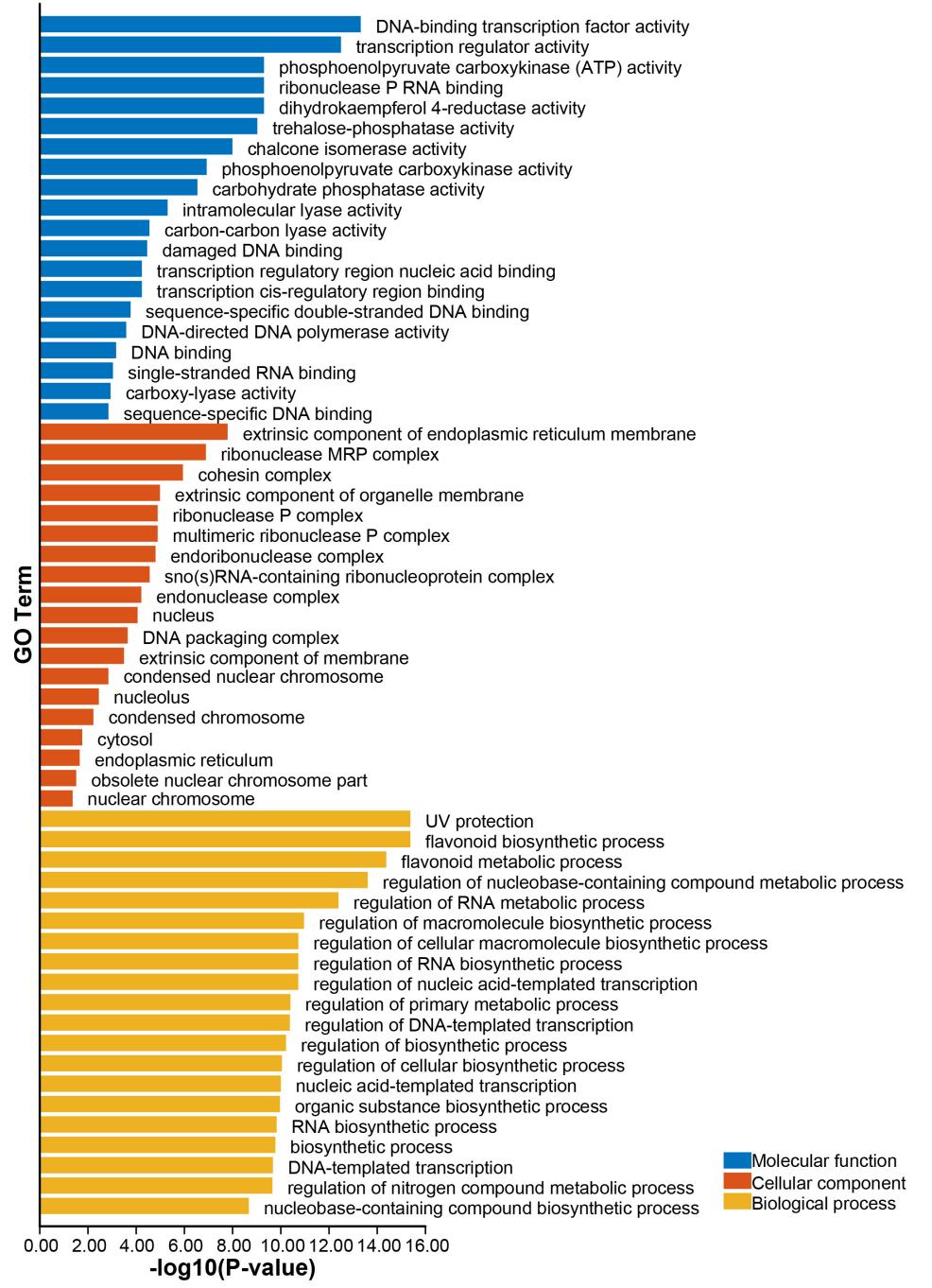


Fig. S4 GO enrichment of the 310 valuable genes


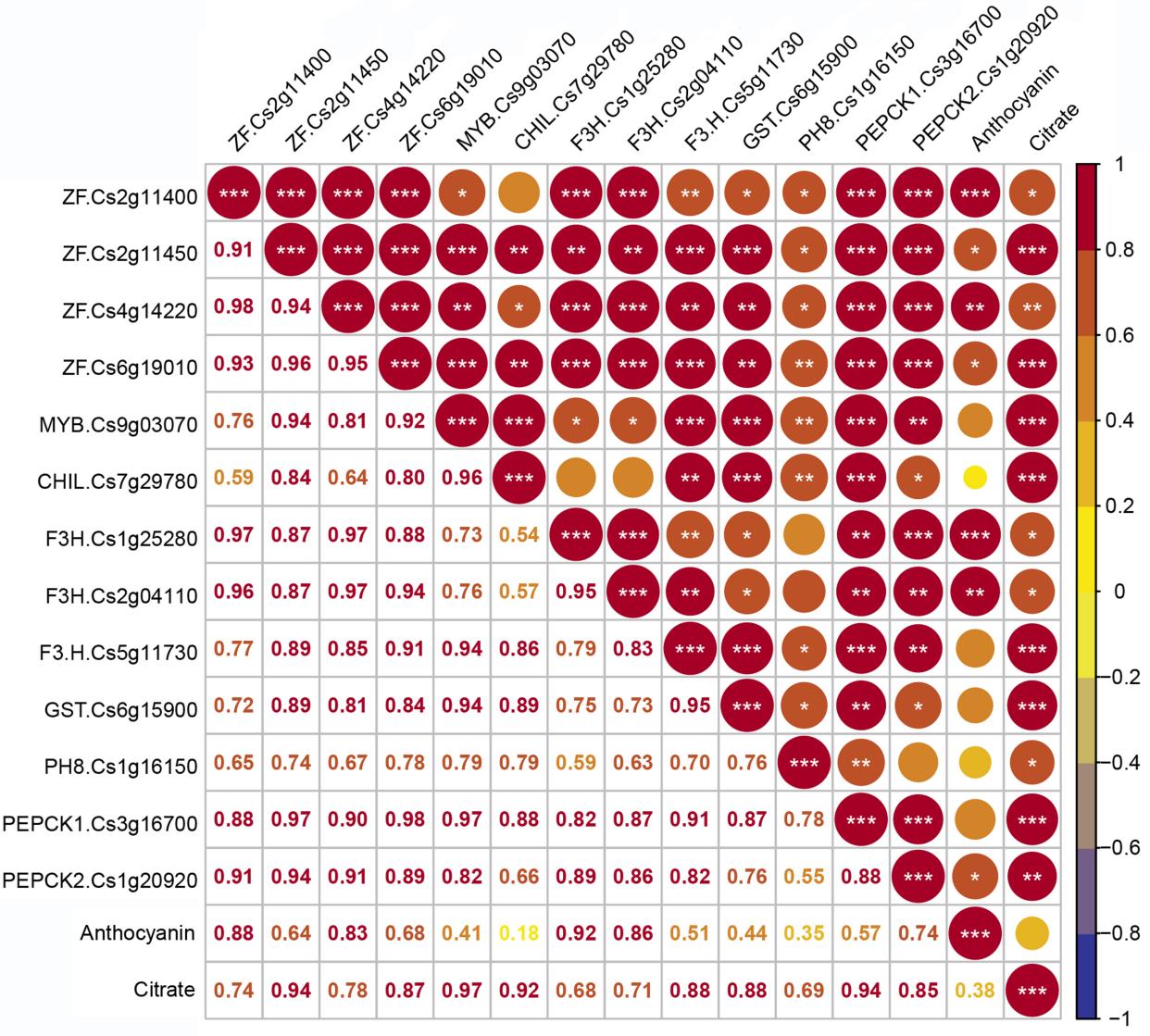


Fig. S5 Correlation analysis of expression profiles of citrate metabolism and anthocyanin biosynthesis related genes, citrate and anthocyanin content. *, ** and *** represent significant differences at *p* < 0.05, *p* < 0.01 and *p* < 0.001, respectively.
